# Acute Depletion of D2 Receptors from the Rat Substantia Nigra Alters Dopamine Kinetics in the Dorsal Striatum and Drug Responsivity

**DOI:** 10.3389/fnbeh.2016.00248

**Published:** 2017-01-19

**Authors:** Evgeny A. Budygin, Erik B. Oleson, Yun Beom Lee, Lawrence C. Blume, Michael J. Bruno, Allyn C. Howlett, Alexis C. Thompson, Caroline E. Bass

**Affiliations:** ^1^Department of Neurobiology and Anatomy, Wake Forest School of MedicineWinston Salem, NC, USA; ^2^Institute of Translational Biomedicine, St. Petersburg State UniversitySt. Petersburg, Russia; ^3^Department of Physiology and Pharmacology, Wake Forest School of MedicineWinston Salem, NC, USA; ^4^Department of Pharmacology and Toxicology, School of Medicine and Biomedical Sciences, University at BuffaloBuffalo, NY, USA; ^5^Research Institute on Addictions, University at BuffaloBuffalo, NY, USA

**Keywords:** D2 autoreceptor, haloperidol, antipsychotics, fast-scan cyclic voltammetry, RNA interference, rat

## Abstract

Recent studies have used conditional knockout mice to selectively delete the D2 autoreceptor; however, these approaches result in global deletion of D2 autoreceptors early in development. The present study takes a different approach using RNA interference (RNAi) to knockdown the expression of the D2 receptors (D2R) in the substantia nigra (SN), including dopaminergic neurons, which project primarily to the dorsal striatum (dStr) in adult rats. This approach restricts the knockdown primarily to nigrostriatal pathways, leaving mesolimbic D2 autoreceptors intact. Analyses of dopamine (DA) kinetics in the dStr reveal a decrease in DA transporter (DAT) function in the knockdown rats, an effect not observed in D2 autoreceptor knockout mouse models. SN D2 knockdown rats exhibit a behavioral phenotype characterized by persistent enhancement of locomotor activity in a familiar open field, reduced locomotor responsiveness to high doses of cocaine and the ability to overcome haloperidol-induced immobility on the bar test. Together these results demonstrate that presynaptic D2R can be depleted from specific neuronal populations and implicates nigrostriatal D2R in different behavioral responses to psychotropic drugs.

## Introduction

Alterations in dopamine (DA) neurotransmission are associated with many neuropsychiatric conditions including schizophrenia, attention deficit hyperactivity disorder and drug addiction. DA uptake via the presynaptic DA transporter (DAT) is essential for controlling synaptic DA concentrations. D2 receptors (D2R) located on presynaptic dopaminergic neurons participate in regulating synaptic DA concentrations by increasing DA uptake (Cass and Gerhardt, 1994; Dickinson et al., 1999; Wu et al., 2002), while also inhibiting DA release (May and Wightman, 1989; Kawagoe et al., 1992; Wu et al., 2002) and synthesis (Budygin et al., 1999a,b) when activated by excessive DA levels. These D2 autoreceptors may participate in DA-related pathologies and represent a potential therapeutic target. However, D2 autoreceptors are difficult to study due to their diverse neuroanatomical localization in multiple brain regions and the presence of D2R on both pre- and post-synaptic sites, as well as the lack of pharmacological reagents that can target each population independently. Being able to separate presynaptic D2 autoreceptor function from postsynaptic D2R in specific brain regions such as the striatum is essential in determining their roles in various neurological conditions.

Until recently, we have not been able to independently manipulate the D2 autoreceptor without also affecting postsynaptic receptors. The first successful effort to genetically target presynaptic D2R was accomplished by Bello et al. (2011) using a floxed-D2R mouse crossed with a strain expressing Cre-recombinase in DAT neurons (*Dat*^+/IRES–cre^). In these animals the D2R were deleted only in dopaminergic neurons while leaving post-synaptic D2R intact. The phenotype of these mice included hyperactivity, increased sensitivities to DAT blockers such as cocaine and methylphenidate, as well as increased sensitivity to typical and atypical antipsychotics including haloperidol and sulpiride, respectively. More recently, Anzalone et al. (2012) used a similar approach by crossing floxed-D2R mice with a mutant strain in which the Engrailed-1 promoter drives Cre expression (En1Cre) in the brain. En1Cre mice express Cre in multiple embryonic sites, including the mid-hindbrain junction in the early embryo (Srinivas et al., 2001), resulting in loss of D2 autoreceptors from projections of the midbrain. The mice demonstrated a phenotype similar to that reported by Bello et al. (2011) with some notable exceptions in presynaptic DA dynamics and responses to dopaminergic drugs. For example, the autoreceptor knockdown line reported by Bello et al exhibited enhanced electrically evoked DA release and no change in its reuptake, while Anzalone et al. (2012) showed an attenuation of DA release and enhancement of reuptake.

Given the role of D2R in fine-tuning DA neurotransmission, it is possible the primary mechanism underlying these discrepancies may be developmental compensation due to chronic hyperdopaminergia, which could obscure the functional role of the D2 autoreceptor. In addition, both mutant strains have a global deletion of D2 autoreceptors in both striatonigral and mesolimbic pathways. Thus having a model in which D2 autoreceptors are deleted acutely in adult animals to avoid developmental compensation, and in a brain region specific manner, could better clarify the role of the autoreceptor in various behaviors and in response to pharmacological agents.

We have pursued a flexible RNA interference approach to study the role of presynaptic D2 autoreceptors in the nigrostriatal pathway of adult, wild type Sprague-Dawley rats. An adeno-associated virus (AAV) was used to deliver a short hairpin RNA (shRNA) targeting the D2R to the substantia nigra (SN). Our data show that depletion of D2R from SN neurons, (hereafter referred to as SN D2 knockdowns) alters DA release and reuptake kinetics in the dorsal striatum (dStr). In addition, the rats display enhanced locomotor activity, suppressed locomotor responses to high, but not low, doses of cocaine and an attenuated cataleptic response to haloperidol. These results demonstrate the utility of this site-specific viral vector approach in enhancing our understanding of how SN D2R participate in regulating DA neurotransmission and responses to dopaminergic agents.

## Materials and Methods

### Materials

**Figure 1:**
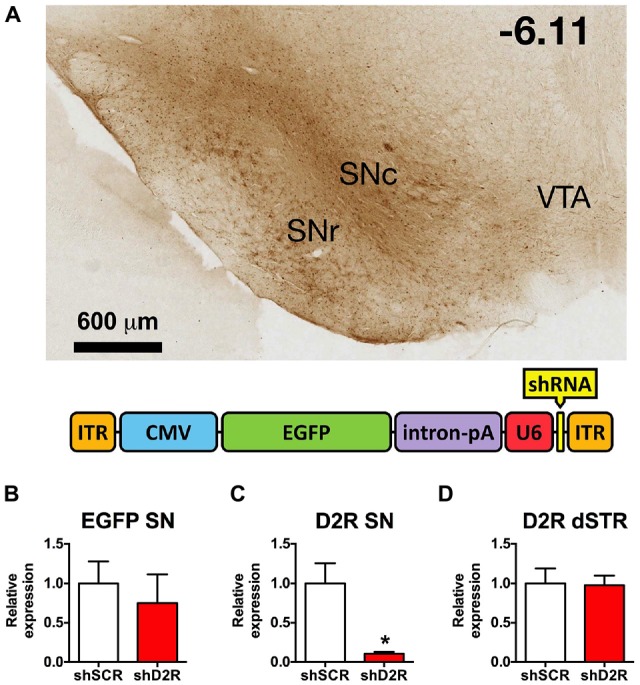
**Knockdown efficiency of D2 receptors (D2R)-shRNA adeno-associated virus (AAV) in the substantia nigra (SN). (A)** Top shows a representative image of EGFP spread in the midbrain visualized with immunohistochemistry for GFP (scale bar equals 600 μm, −6.11 mm from bregma). Bottom is a graphic depiction of the transgene contained in the AAV (ITR, inverted terminal repeat; CMV, cytomegalovirus promoter; EGFP, enhanced green fluorescent protein; intron/pA, combined intron and poly adenylation sequence; U6, mouse U6 promoter; shRNA, short hairpin RNA). **(B)** Relative EGFP mRNA levels as determined by real time qPCR of SN injected with scrambled (SCR) control or D2 shRNA-AAV (*n* = 4–6). **(C)** Relative D2R mRNA levels in the SN and **(D)** dorsal striatum (dSTR), *p < 0.05.

All experiments and procedures were approved either by the Wake Forest School of Medicine or University at Buffalo, SUNY Institutional Animal Care and Use Committees, followed the guidelines of the NIH Guide for the Care and Use of Laboratory Animals, and comply with the ARRIVE guidelines. Adult male Sprague-Dawley rats weighing between 250–300 g were obtained from Harlan Laboratories (Frederick, MD, USA). Animals were housed two to a cage with food and water available *ad libitum*. The facilities are under a standard 12/12 h light dark schedule with lights off at 03:00 h and on at 15:00 h. All procedures were conducted between the hours of 10:00 h and 13:00 h. *Viral Constructs*: the shRNA-AAV plasmids were cloned as previously described (Sadri-Vakili et al., 2010; Lazarus et al., 2011). The enhanced green fluorescent protein (EGFP)-U6-pACP plasmid contains AAV2 inverted terminal repeats (ITR) flanking a cytomegalovirus promoter (CMV), the EGFP gene, an intron and polyadenylation signal derived from SV40, and further downstream, a murine U6 pol III promoter (mU6, Figure 1A). Briefly, synthetic oligos encoding the shRNA and its respective complement (Integrated DNA Technologies, Coralville, IA, USA) were annealed and ligated into unique BbsI and NheI sites downstream of the U6 promoter. Three shRNAs were designed to target different regions of the rat D2 mRNA (NM_012513). It should be noted that the shRNA targets are found in both D2 short and D2 long receptor isoform mRNA. The control vector, shSCR-AAV, encodes EGFP plus a scrambled (SCR) shRNA, which does not correspond to any known rat mRNA sequence. Packaging of all AAV using standard transfection protocols to generate helper virus-free pseudotyped AAV2/10 virus (Xiao et al., 1998) has been described elsewhere (Bass et al., 2010). The vector stocks were titered by real-time quantitative PCR using the ABI Prism 7700 Sequence Detection System from Perkin-Elmer Applied Biosystems (Foster City, CA, USA) as previously described (Clark et al., 1999). The average titer of the preparations was approximately 1 × 10^12^ vector genomes/ml. For all experiments, the three D2 shRNA viruses were mixed and co-injected. Previous studies have demonstrated that the co-administration of shRNAs with mixed knockdown efficiencies can produce more than additive effects and a greater knockdown than an individual shRNA alone (Bahi et al., 2005). We have previously demonstrated the effectiveness of these viruses in knocking down post-synaptic D2R in rat dStr (Blume et al., 2013).

### Stereotaxic Injections

Rats were anesthetized with 80 mg/kg ketamine and 12 mg/kg xylazine (i.p.), placed in an adult rat stereotaxic frame (Kopf Instruments, Tujunga, CA, USA) and an incision was made along the midline of the scalp exposing bregma. Burr holes were drilled over the injection site and a 10 μl syringe (Hamilton Company, NV, USA) was inserted vertically above the SN (AP −5.6 mm, ML ±2.0 mm, DV −7.6 mm). Animals were injected with 0.5 μl D2R shRNA-AAV over a 5 min period with an infusion rate ~0.1 μl/min. The needle was maintained in place for an additional 5 min and then slowly withdrawn. The incision was sutured and rats returned to their home cage.

### Confirmation of Viral Transduction

Rats were deeply anesthetized and transcardially perfused with 10% formalin. The brains were removed, incubated in 10% formalin overnight (4°C) while shaking, followed by at least 24 h in a 25% sucrose/phosphate buffered saline (PBS) solution. Free-floating sections (40 μm) were obtained and stored in 0.2% sodium azide-PBS at 4°C. To confirm placement and viral spread, floating sections were either mounted in Prolong Gold media (Invitrogen) to analyze native EGFP fluorescence, or processed for GFP immunohistochemistry. Tissue sections were incubated with a 1% H_2_O_2_ phosphate buffered saline-tween20 (PBST) solution for 30 min, rinsed three times with PBS, and incubated with a rabbit anti-GFP antibody (Invitrogen, catalog no. A6455) 15–18 h at room temperature. Sections were rinsed three times with PBS and incubated with biotin-SP-conjugated AffiniPure Donkey anti-Rabbit IgG (H + L) (Jackson ImmunoResearch Labs, cat. no. 711-065-152) for 1 h. After rinsing another three times with PBS, the sections were incubated with ABC reagent (Vectorlabs cat. no. PK-6100), rinsed another three times with PBS and stained with diaminobenzidine (DAB, Sigma). Sections were then mounted and coverslipped with Permount. Only rats that exhibited correct placement with viral spread restricted primarily to the SN were included in the analyses, while rats demonstrating spread overlapping with the VTA were excluded.

### Real-Time Quantitative PCR

A cohort of rats was unilaterally infused with 1.0 μl of D2R (*n* = 6) or SCR shRNA virus (*n* = 4) in the SN and sacrificed by decapitation 2–4 weeks later under deep anesthesia induced with ketamine and xylazine. Two mm coronal brain punches were taken at both the injection site and the contralateral uninjected side. Total RNA was isolated from each punch with an RNeasy Micro Kit (Qiagen, Hilden, Germany), eluted with 14 μl of ribonuclease-free water and stored at −80°C. RNA quantification and purity was determined with purity set at a 260/280 ≥ 2.0 and 260/230 ≥ 1.8 (NanoDrop 1000 Spectrophotometer, Thermo Scientific, DE, USA). Total RNA (500 ng) was reverse transcribed into cDNA using a High Capacity cDNA Reverse Transcription Kit (Applied Biosystems, CA, USA) according to the manufacturer’s instructions. Real-time qPCR was performed in triplicate for each sample using a Bio-Rad Miniopticon Real-Time PCR system (Bio-rad, Hercules, CA, USA). Sybr assays were used to determine the expression of the following genes: rat Enolase 2 (*ENO2*), rat D2 dopamine receptor (*DRD2*), rat beta-actin (*BACT*) and EGFP. The following primer sets were used in these experiments. *ENO2*: ACGTCTGGCGAAGTACAACC (forward), GTCGGGACAGCAAGAAAGAG (reverse), *BACT*: CATCCTGCGTCTGGACCTGG (forward), TAATGTCACGCACGATTTCC (reverse). The *DRD2* and tyrosine hydroxylase *(TH)* genes was quantified using the Bio-Rad PrimePCR^TM^ Sybr^®^ Assay (qRnoCID0001883 and qRnoCID0002299, respectively). The stably expressing housekeeping gene *BACT* was used as a reference genes and relative quantification of gene expression levels was assessed using the comparative CT method (2^−ΔΔCT^ method; Livak and Schmittgen, 2001). SN punches that did not contain GFP were considered a missed infusion and not included in the analyses. The difference between the means was evaluated by a Student’s *t*-test and differences at the *P* < 0.05 level were considered significant.

### Fast Scan Cyclic Voltammetry (FSCV)

A cohort of rats was injected with either the shD2R-EGFP-AAV10 or shSCR control virus in the SN and 2 weeks later the ability of haloperidol to alter DA release and reuptake was determined using voltammetry. While under urethane anesthesia (1.5 g/kg, i.p.), rats were secured in a stereotaxic frame in a flat skull position. Holes were drilled into the skull above the striatum for the detecting electrode (AP: +1.3, ML: +1.5 (±0.2) in mm relative to bregma) and SN for the stimulating electrode (AP: −5.6, ML: +2.0 mm). An additional hole was drilled into the skull of the contralateral hemisphere into which an Ag/AgCl reference electrode was implanted just below the surface of the skull. A carbon fiber microelectrode (approximately 80–200 μm in length beyond the glass capillary in which it was contained) was secured to the stereotaxic frame arm and also connected to the amplifier. The carbon fiber electrode was placed into the hole above the striatum, and lowered approximately 5 mm from the surface into the striatum. Voltammetric recordings were made at the recording electrode every 100 ms for a 15 s duration by applying a triangular waveform (−0.4 to +1.2 V, 400 V/s). The biphasic stimulation applied by the stimulating electrode consisted of 60 rectangular pulses at 60 Hz, 300 μA and was activated at 5 s into each recording. Recorded signals showed an oxidation peak at +0.6 V and a reduction peak at −0.2 V (vs. Ag/AgCl reference), ensuring that the released chemical was indeed DA. The carbon fiber microelectrode and bipolar stimulating electrode were independently positioned at depths of 4.6–5.2 mm and 7.5–8.3 mm, respectively, to optimize the amplitude of electrically evoked DA release. Once evoked DA recordings were optimized, stable readings were collected every 5 min for at least 20 min. When baseline recordings were within 10% of each other for at least five measurements, haloperidol or saline was injected. Stimulated recordings were then made at 1, 5 and 10 min and thereafter every 10 min up to 120 min following drug injection. Data were digitized (National Instruments, Austin, TX, USA) and stored on a computer. Carbon fiber microelectrodes were post-calibrated *in vitro* with known concentrations of DA (2–5 μM). Calibrations were performed in triplicate and the average value for the current at the peak oxidation potential was used to normalize recorded *in vivo* current signals to DA concentration. DA uptake was determined from the clearance rate of DA and was assumed to follow Michaelis-Menten kinetics. The changes in DA during and after electrical stimulation were fit using the equation:

$$\text{d}\left[\text{DA}\right]/\text{d}t\;=\;\left(\left(f){\left[\text{DA}\right]}_{\text{p}}-(V_{\text{max}}/\{(K_{\text{m}}/\left[\text{DA}\right])+\text{1}\}\right)\right)$$

where *f* is the stimulation frequency (Hz), DA release per pulse [DA]_p_ is the concentration of DA released per stimulus pulse, and *V*_max_ is the maximal rate of DA uptake, which is proportional to the number of DAT proteins. The baseline value of *K*_m_ was calculated to be between 0.16–0.2 μM, a value determined in rat brain synaptosomes and co*mm*only used in the analysis of voltammetric data (Near et al., 1988; Garris and Rebec, 2002). The derivative form of the above equation was used to simulate the DA response (Garris et al., 1997; Budygin et al., 1999a; Wu et al., 2001; Garris and Rebec, 2002; Oleson et al., 2009a,b). Rats were injected with saline, 0.5 or 1.0 mg/kg haloperidol i.p. (*n* = 5 per dose) and the effects on DA release and reuptake measured.

### Behavioral Tests

Rats serving to assess the effect of SN D2 knockdown on behavior were injected with 0.5 μl virus as described above. The experimenter was blind to which virus the rats received. The first cohort of rats was acclimated to the open field chambers (17 × 17 × 12 inch, Med-Associates) for 1 h and total distance traveled (in cm) was recorded during 12, 5-min consecutive bins for three consecutive days prior to surgery. One week after surgery locomotor activity was again measured and subsequently tested once a week for a total of 8 weeks post-surgery. Another cohort was challenged 4 weeks after viral infusions in a Latin square design with saline, 0.5, 1.0 or 2.0 mg/kg haloperidol i.p., to evaluate the effects of haloperidol induced catalepsy using the bar test. Each rat received only one dose per day with 3 days between doses. One hour after injection the front paws of the rats were placed on a metal bar (1 cm in diameter, positioned 9 cm above the surface) and the amount of time until both paws were removed and returned to the table surface was measured, with a 3 min maximum time allowed. After an additional week for washout the rats were placed in the open field chambers and following 30 min of acclimation challenged with saline, 5, 10 or 20 mg/kg cocaine i.p. in an escalating dose design to evaluate the entire dose-response curve, with challenges occurring at 4 day intervals. Data are presented as total distance traveled (m), and % of saline response, where % saline response = m traveled cocaine/saline × 100. An ascending dose response was chosen for cocaine in an effort to minimize the effects of locomotor sensitization.

### Statistical Analyses

The data are presented as mean ± SEM and the criterion of significance was set at *p* < 0.05. Real-time qPCR, voltammetry and behavioral data were analyzed using *t*-test, one-way repeated measures or two- way ANOVAs with a Bonferroni and Simple Effects post-tests, respectively, to determine statistical significance.

## Results

### Site Specific Depletion of D2R from SN Neurons and their Projections

In previous studies we established that the D2R shRNA viruses significantly decrease both the mRNA and protein levels of the post-synaptic D2R when injected directly into the dStr (Blume et al., 2013). Here we demonstrate that D2R mRNA levels are likewise significantly decreased after D2R shRNA-AAV infusions into the SN. Immunohistochemistry for EGFP, which is also produced by the D2R and SCR viruses, reveals the extent of transduction. In Figure 1A we show the EGFP expression in the SN from a representative rat 8 weeks after it was injected with the D2R shRNA virus. EGFP expression was largely restricted to the SN, and while both SN pars compacta (SNc) and SN pars reticulata (SNr) were transduced, the SNc had more prominent/dense signal. Missed infusions including spillover into the VTA were excluded from all analyses. We have observed detectable levels of EGFP in the SN within 3 days of infusing the virus, with maximal expression achieved within 1–2 weeks post-infusion, and expression lasting for at least 4 months (data not shown). Expression of EGFP in the terminals is also observed in this time frame. Real time qPCR analysis revealed an ~90% reduction of D2R RNA expression when comparing rats infused with D2 shRNA virus (Figure 1B) and those infused with the SCR control virus (Figure 1C). Since AAV10 will transduce primarily the neuronal cell bodies at the site of the injection (Cearley and Wolfe, 2006), injection of the virus directly into the SN will produce a knockdown in any neuronal cell body that expresses D2 mRNA in this region, including dopaminergic neurons. The D2 autoreceptor on dopaminergic terminals projecting from the SN to the dStr as well as the somatodendritic D2 autoreceptors should be affected. As long as the virus does not spread to the VTA, the D2 autoreceptor population on the cell body and projections for this population of DA neurons will be spared. In addition, we observed no changes in D2 mRNA levels in dStr neurons (Figure 1D).

### Knockdown of SN D2R Alters DA Dynamics in the Dorsal Striatum and Attenuates the Ability of Haloperidol to Enhance DA Release

**Figure 2:**
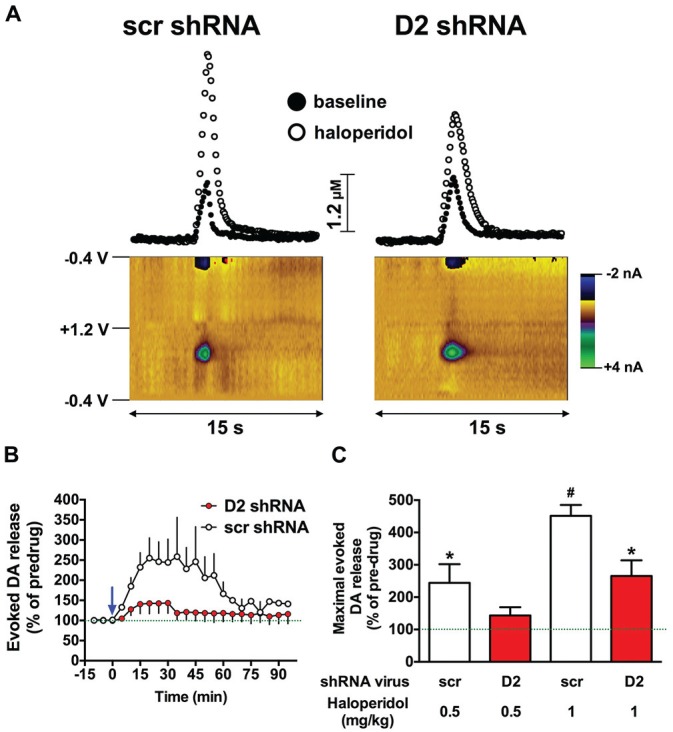
**SN D2 autoreceptor knockdown blocks haloperidol induced enhancement of electrically evoked dopamine (DA) release. (A)** Left panels display fast scan cyclic voltammetry (FSCV) data from a representative SCR virus control animal while panels on the right are from a representative SN D2 autoreceptor knockdown rat. Measurements were taken in the dStr over a 15 s period. Top panels demonstrate concentrations vs. time traces extracted from the data at 0.65 V; the peak oxidation for DA. Bottom panels represent standard color plots, which topographically depict the voltammetric data before haloperidol injection with time on the *x*-axis, applied scan potential on the *y*-axis and background-subtracted faradaic current shown in the *z*-axis in pseudo-color. The identical color plots were obtained following haloperidol administration with the exception that background-subtracted current at 0.65 V was noticeably stronger. **(B)** Represents a time course of electrically evoked DA release normalized to pretreatment amounts (first three measurements). The mean electrically evoked DA release (% of evoked DA release in pre-haloperidol measurements) ± SEM are graphed (*n* = 8–10), measurements were taken every 5 min for 1.5 h. The blue arrow indicates administration of 0.5 mg/kg haloperidol immediately after the third baseline measurement was taken. **(C)** Demonstrates the mean maximal electrically evoked DA release ± SEM achieved in the 90 min after 0.5 or 1.0 mg/kg haloperidol i.p. Data were analyzed with one-way ANOVA followed by Bonferonni *post hoc* tests. Dashed line indicates 100% of pre-drug levels, *indicates a significant difference from the rats pre-drug baseline measurements (*p < 0.05), while # indicates a significant difference from baseline as well as the corresponding SN D2 autoreceptor knockdown rats treated with the same dose of haloperidol (^#^*p* < 0.05).

Haloperidol enhances electrically-evoked DA release in the striatum, primarily by blocking presynaptic D2 autoreceptors, which disinhibits DA release and attenuates reuptake through the DAT (Garris and Rebec, 2002; Garris et al., 2003). Therefore, we hypothesized that knockdown of the SN D2 autoreceptor would blunt haloperidol-induced increases in an electrically-evoked DA release in the dStr. Two weeks after infusion of the D2 shRNA-AAV into the SN, we assessed the effects of haloperidol on DA release in the dStr using fast-scan cyclic voltammetry. We chose an early time point in an effort to avoid consequences of neurochemical compensation due to the loss of the D2R over time. As predicted, 0.5 mg/kg of haloperidol induced a significant increase in electrically-evoked DA release in SCR treated control rats, and this effect was attenuated in the SN D2 knockdown animals (*F*_(1,60)_ = 27.42, *p* < 0.0001; two-way ANOVA). Figure 2A shows color plots from representative SCR control and SN D2 knockdown rats, prior to treatment with 0.5 mg/kg haloperidol i.p. The time course of 0.5 mg/kg haloperidol on electrically evoked DA release is presented in Figure 2B. When normalized to baseline levels (% predrug), 0.5 mg/kg haloperidol significantly increased maximal evoked DA release in SCR controls (Bonferroni’s *p* < 0.05), an effect that was absent in SN D2 knockdown rats (Figure 2C). When a higher dose of 1.0 mg/kg haloperidol was tested both groups of rats exhibited a significant increase in electrically evoked DA release compared to their predrug baseline; however, the increase was greatly attenuated in SN D2 knockdown rats compared to SCR controls, and these two groups were significantly different from one another (Bonferroni’s *p* < 0.05).

**Figure 3:**
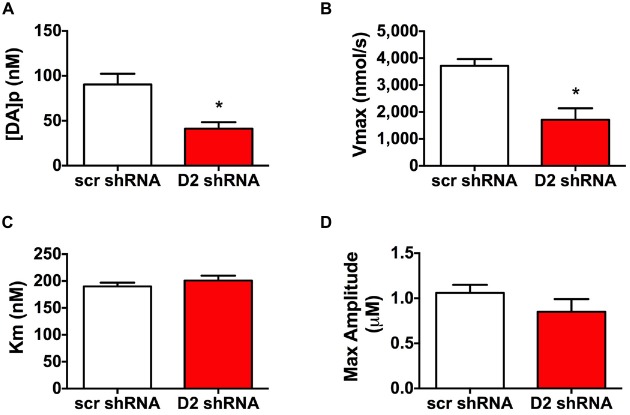
**Altered DA release and reuptake kinetics in SN D2 autoreceptor knockdown rats.** Electrically evoked DA release during baseline measurements were analyzed and the mean ± SEM kinetic parameters of **(A)** DA per pulse, **(B)** *V*_max_, **(C)** *K*_m_ and **(D)** maximal amplitutes (μM) are graphed (*n* = 8–10, *p < 0.05).

To evaluate DA release and uptake changes following SN D2 knockdown we used kinetic analysis of the baseline electrically evoked fast-scan cyclic voltammetry data, based on the neurochemical model developed by Wightman (Near et al., 1988). As described previously, the analysis evaluates measured electrically-induced DA efflux in terms of one parameter for DA release and two parameters of DA uptake. These parameters were determined by fitting experimental results to curves simulated by the model (Bass et al., 2010). The analysis of the electrically evoked DA signals indicated that [DA]_p_ was significantly decreased following SN D2 knockdown (*p* < 0.005, *t* = 3.321, df = 16; Unpaired *t* test; Figure 3A). Similar changes were found with the parameter of DA uptake, *V*_max_, which decreased from 3715 ± 255 nmol/s in SCR control rats to 1711 ± 428 nmol/s in SN D2 knockdown rats (*p* < 0.005, *t* = 4.024, df = 14; Unpaired *t* test; Figure 3B). At the same time, no significant alterations in the second Michaelis-Menten parameter for DA uptake, apparent *K*_m_, were seen (190 ± 7 vs. 201 ± 9 nM for SCR control and D2 knockdown group, respectively, Figure 3C). Maximal amplitude of electrically-evoked DA efflux was also not affected by SN D2 knockdown (1.06 ± 0.09 vs. 0.85 ± 0.14 μM, Figure 3D) which is in agreement with proportional changes in [DA]_p_ and *V*_max_. Together these data indicate that loss of the D2 autoreceptor from SN terminals in the dStr attenuates the ability of haloperidol to enhance DA release, and that a consequence of D2 autoreceptor depletion is a decrease in *V*_max_ or reuptake by approximately 56%.

### Depletion of D2R from the SN Is Associated with Locomotor Hyperactivity and Loss of Habituation

**Figure 4:**
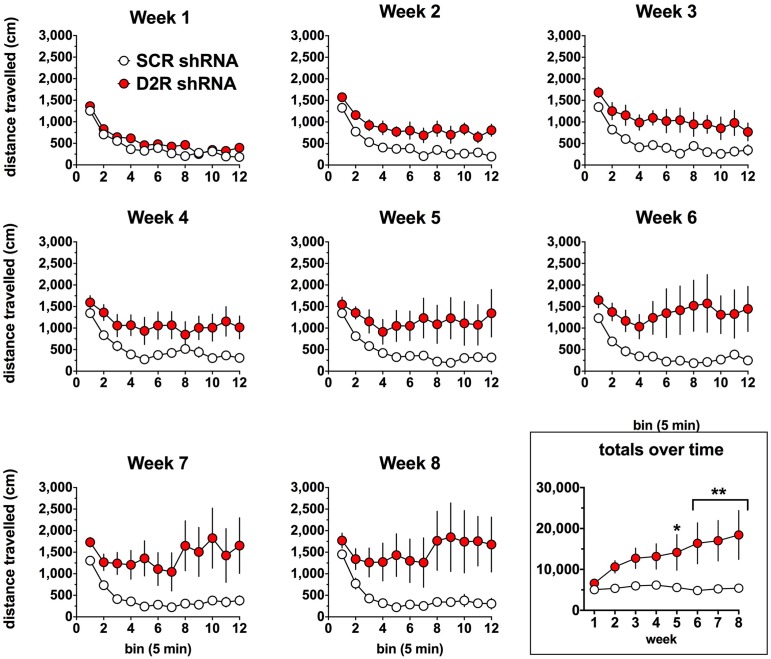
**Effect of SN D2 autoreceptor knockdown on spontaneous locomotor activity.** Each panel represents a 1-h session in which distance traveled was measured in 5-min bins. Circles represent the mean ± SEM of either D2 shRNA-AAV (red) or SCR shRNA control AAV (white) injected rats. The rats were tested weekly after virus infusion on the same day of the week and at the same time of day. Data were analyzed by comparing total distance traveled (last panel) by each virus group over each post-injection week using a repeated measures two-way ANOVA. The last panel on the lower right shows the total distance traveled for the entire 1-h weekly sessions. *Indicates a significant difference with *p* < 0.05, **p < 0.01, ***p < 0.001 and ****p < 0.0001.

We next examined the effect of SN D2 knockdown on locomotor activity over time. Rats were allowed 1 week to recover before testing in an open field activity chamber and were assessed each week thereafter for 2 months (Figure 4). Analysis of the total distance traveled in the eight weekly sessions revealed a significant interaction between virus and week (two-way repeated measures ANOVA, *F*_(7,98)_ = 3.53, *P* = 0.002). *Post hoc* analysis using Simple Effect tests (Winer, 1962) showed a significant increase in total distance traveled in the SN D2R knockdown rats starting at week 2 and no change in total distance traveled each week in SCR controls over the 8 week period. Total distance traveled by SN D2R knockdown rats became significantly greater than that of SCR controls at 5 weeks and continuing through 8 weeks, the last time point measured (for Simple Effect test of Virus at each week, see Table 1). A *post hoc* exploratory analysis was conducted on bin data during weeks 5–8 to further explore the pattern of change in locomotor activity over the 60 min session and to test the idea that SN D2R knockdown might modify the pattern of within session habituation. A two-way ANOVA comparing locomotor activity by 5-min bins (averaged over weeks 5–8) between SCR and SN D2R knockdown rats found a significant interaction between virus and bin (Simple Effects Test, *F*_(11,154)_ = 2.36, *p* = 0.01) in which differences in locomotor activity between SN D2 knockdown rats and SCR controls occurred in bin 5 and bins 7–12 only (Simple Effects tests, *p* < 0.05, see Table 2). The magnitude of the difference increased with time to peak at bin 9 and then leveled off. Among SCR controls, and as expected, there was a significant decrease in locomotor activity across bins (Simple Effects test comparing bins in SCR, *F*_(11,154)_ = 5.56, *p* < 0.0001); locomotor activity decreased significantly in bin 1, 2 and 3 and then leveled off. Among SN D2 knockdown rats, no significant difference in locomotor activity by bin was observed (Simple Effects test, *F*_(11,154)_ = 1.81, *p* = 0.0695). The pattern of these results suggests that the change in locomotor activity consequent to SN D2 knockdown lies in part in the reduction or absence of within session habituation. The hyperactivity exhibited by the SN D2R knockdown rats is similar to that reported for global D2 autoreceptor knockdown mice by Bello et al. (2011). However, in the Bello et al. (2011), study both wildtype and D2 autoreceptor knockouts demonstrated a progressive decrease in distance traveled with each successive bin within a session as the mice habituated to the chamber. In our model, we saw a similar pattern in both the SCR and D2R shRNA rats during the first 4 weeks, but with subsequent weeks the shape of this curve began to change in SN D2 knockdown rats, in which the typical decrease in locomotor activity with successive bins during the session either reversed or was absent (Figure 4).

**Table 1:** **Summary of spontaneous locomotor activity**.

	Mean total distance traveled (cm) In 1 h ± SEM		
Time (weeks)	SCR control shRNA	D2R shRNA	*F*_(1,23)_
Baseline	6152 ± 415	6269 ± 422	
1	5049 ± 402	6602 ± 505	<1
2	5367 ± 573	10621 ± 1520	1.72
3	5976 ± 736	12724 ± 2478	2.89
4	6169 ± 793	13177 ± 3088	3.11
5	5578 ± 639	14149 ± 4352	4.65*
6	4842 ± 660	16394 ± 5005	8.46**
7	5254 ± 650	17012 ± 4955	8.47**
8	5422 ± 865	18435 ± 5981	10.73**

*Significant difference with *p* < 0.05 and ** *p* < 0.01.

**Table 2:** **Summary of Locomotor activity (average week 5–8)**.

Bin	SCR	SN D2 KD	*F*_(1,19)_	*p*
1	1333^t^	1675	<1	NS
2	752^t^	1334	1.58	0.22
3	471^t^	1206	2.53	0.128
4	360	1106	2.61	0.1227
5	283	1269	4.56	0.046*
6	287	1201	3.93	0.0621
7	271	1237	4.39	0.0498*
8	266	1505	7.21	0.0147*
9	259	1539	7.69	0.0121*
10	344	1497	6.36	0.0208*
11	343	1396	5.2	0.0343*
12	314	1530	6.94	0.0163*

^t^Significantly different than all other bins in that group (*p* < 0.05). *Significant difference between SCR and SN D2 KD (*p* < 0.05).

### Decreased Haloperidol-Induced Catalepsy in SN D2 Knockdown Rats

**Figure 5:**
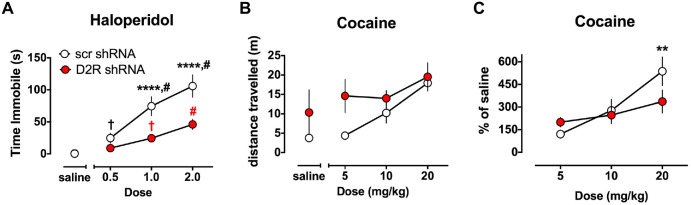
**Altered sensitivity to dopaminergic drugs in SN D2 knockdowns. (A)** Catalepsy induced by the D2 antagonist haloperidol was measured as time immobile in the bar test. Circles represent the mean amount of time immobile ± SEM. ^†^Significantly different from saline *p* < 0.05, # significantly different from all other doses *p* < 0.01, *** significantly different between virus groups at *p* < 0.0001. Black and red marks correspond to the scr and D2R shRNA data points, respectively. In a separate cohort of animals, the ability of the DA transporter (DAT) blocker cocaine to enhance locomotor activity was tested and measured as distance traveled **(B)** and transformed as percent of saline **(C)**. With both drugs a dose response was conducted in either a randomized order (haloperidol) or in an ascending fashion (cocaine) with 3 days between doses to allow for elimination of the drug. Circles represent the mean distance traveled ± SEM **(B)**, or % of saline distance traveled **(C)** of either D2 shRNA-AAV (red) or SCR shRNA control AAV (white) injected rats (*n* = 5–11). *Indicates a significant difference with *p* < 0.05, **p < 0.01, ***p < 0.001 and ****p < 0.0001.

The ability of haloperidol to induce catalepsy was tested in a separate cohort of SN D2 knockdown rats 7 weeks after virus infusion into the SN. As indicated in Figure 5A, SCR control virus injected rats exhibited dose dependent increase in the time needed to remove both paws from the bar and return them to the table surface. However, the ability of haloperidol to elicit this response was reduced in SN D2 knockdown rats. Two-way ANOVA with repeated measures on dose found a significant interaction between dose and virus (*F*_(3,54)_ = 7.724, *p* = 0.0002) and a significant main effect of each variable (dose *F*_(3,54)_ = 43.78, *p* < 0.0001, virus *F*_(1,18)_ = 14.15, *P* = 0.0014). *Post hoc* tests (Simple Effect tests) of the effect of dose in SCR control rats (*F*_(3,54)_ = 39.8, *p* < 0.0001) showed that haloperidol significantly increased the time on the bar at each dose (in mg/kg, 0.0 < 0.*5* < 1.0 < 2.0 by Student Neumann-Keuls pairwise comparison test). In contrast, the effect of dose in SN D2 knockdown rats (*F*_(3,54)_ = 8.50, *p* < 0.001) showed that haloperidol significantly increased time on the bar at 1.0 and 2.0 mg/kg doses only (in mg/kg, 0 = 0.05 and 0 < 1.0 < 2.0). Furthermore, time on the bar was significantly reduced in SN D2 knockdown rats relative to SCR control rats at the two highest doses of 1.0 (*F*_(1,63)_ = 18.9, *p* < 0.0001) and 2.0 (*F*_(1,63)_ = 27.0, *p* < 0.0001) mg/kg doses only.

Even though the behavioral assay demonstrated less catalepsy at each dose there were some qualitative haloperidol-induced behavioral deficits that the experimenter could distinguish from vehicle injected subjects. Abnormal rigid postures, associated with akinesia, were often observed in the home cage in both the SN D2 knockdowns and SCR controls after receiving haloperidol. However, the D2 knockdowns that displayed rigidity in their home cage were mobile when placed on the bar and quickly returned to four paws, however they did so with uncoordinated, erratic movements.

### Knockdown of Dorsal Striatal D2 Receptors Alters Responsiveness to Cocaine Induced Locomotor Effects

The ability of cocaine to enhance locomotor activity was assessed in SN D2 knockdown rats and SCR controls using an escalating dose paradigm, with 4 days between challenge cocaine doses. As expected, SCR virus treated rats exhibited a dose responsive increase in locomotor activity when challenged with cocaine (Figure 5B). Repeated measures two-way ANOVA found a significant main effect of dose (*F*_(3,24)_ = 9.667, *p* = 0.0002), no main effect of virus (*F*_(1,8)_ = 2.509, *p* = 0.1519), and no significant interaction between dose of cocaine and virus, (*F*_(3,24)_ = 1.327, *p* = 0.2890). However, it was noted that there was substantial variability in the baseline locomotor response among the rats (i.e., locomotor response to a saline injection), and no significant group differences (*F*_(1,8)_ = 1.264, *p* = 0.293) to which this variability could be ascribed. Therefore, we transformed the data to a percent of saline response to constrain the variability (Figure 5C). When normalized in this fashion, a significant interaction between virus and dose of cocaine is found (*F*_(2,16)_ = 4.33, *p* = 0.031). Simple Effect tests were used to probe the interaction and showed that the locomotor response of SN D2 knockdown rats to 5 mg/kg cocaine is not significantly greater than SCR control rats (*F*_(2,17)_ < 1.0), but that the response to 20 mg/kg dose is significantly less in SN D2 knockdown rats than SCR control rats (*F*_(2,17)_ = 6.08, *p* < 0.01). This pattern of change was also noted in the analysis of dose by virus which revealed that although there was an effect by dose in the SCR control rats (*F*_(2,16)_ = 19.23, *p* < 0.0001), there was no effect of dose in the SN D2 knockdown rats (*F*_(2,16)_ = 2.08, *p* = 0.1574).

## Discussion

The role of the presynaptic D2 autoreceptor in the regulation of striatal DA neurotransmission in distinct circuitries (nigrostriatal vs. mesolimbic) is not completely understood, due in large part to the lack of dopaminergic reagents that can independently modulate the pre- and postsynaptic D2R pools. Thus, a genetic approach is necessary to achieve specificity in targeted manipulations of the autoreceptor. We have previously shown that RNAi is an effective approach to manipulate D2R subpopulations, by specifically knocking down D2R expression in the dStr (Blume et al., 2013). Here we demonstrate for the first time depletion of the D2 from SN neurons exclusively without also manipulating the post-synaptic D2R, or D2R in the VTA and on mesolimbic dopaminergic projections.

Two D2 autoreceptor knockout mouse strains have been created recently; however, these strains have the D2 autoreceptor deleted globally throughout the entire brain and from conception, which limits understanding of regional contributions and is confounded by the potential for developmental neuroadaptations. In addition, there are distinct differences in the phenotypes of the two D2 autorecpetor knockout (D2autoKO) transgenic mice generated thus far. For example, the mice produced by Bello et al. (2011) exhibited no change in DA uptake, an increase in electrically evoked DA release and hyperactivity in novel and familiar environments (Bello et al., 2011), while the Anzalone et al mutant produced opposite results including increased DA uptake, decreased electrically evoked release and hyperactivity only in novel environments but not the farmiliar home cage environment (Anzalone et al., 2012). As the authors in the Anzalone study pointed out, one possible explanation for these discrepancies is that the *Dat*^+/IRES–cre^ line used to generate the Bello DAT-D2autoKO mice likely produce less Cre in cortical regions, as these areas naturally have less DAT expression (Freed et al., 1995; Sesack et al., 1998). Therefore, the resulting mutants may potentially retain some D2 autoreceptor in these cortical regions. In addition, the *Dat*^+/IRES–cre^ line also has reduced expression of the DAT overall compared to wildtype mice, potentially further confounding DA neurotransmission experiments with this resulting Bello mutant (Bäckman et al., 2006). However, it is important to note that the En1Cre used in the Anzalone study is not well characterized in the literature, and may have incomplete or ectopic expression issues as well. In addition, the global nature of the knockout in both studies, from numerous cortical and subcortical regions, undoubtedly confound the underlying regional contributions of the D2 autoreceptor to a particular behavior. The current study uses RNA interference to manipulate D2R expression specifically in SN neurons of adult rats. The specific advantage of RNAi is the ability to manipulate pre- and postsynaptic receptor pools from discrete brain regions in adult, wildtype animals, thereby avoiding developmental compensation and allowing mapping of the effect to a discrete receptor pool. In addition, depletion of the receptor, vs. the total deletion in a knockout, allows us to study graded responses.

In an attempt to understand how depletion of the D2 autoreceptor affects DA neurotransmission in the dStr, we injected the D2 shRNA virus in the SN and examined electrically evoked DA release and reuptake kinetics in the dStr. Analysis of DA kinetics revealed pronounced alterations in electrically evoked DA signals, including a decrease in [DA] per pulse and *V*_max_ (Figures 3A,B), while *K*_m_ was not affected (Figure 3C). Our observations are in sharp contrast to the transgenic D2 autoreceptor knockout mutants, in which DAT uptake was not altered (DAT-D2autoKO mouse) or enhanced DA reuptake was observed (Eng1-D2autoKO mouse). An obvious explanation for these discrepancies between models are species differences in the neuroanatomy, DA kinetics or D2 expression levels between mice and rats. It is also plausible that these discrepancies in DAT function between our acute genetic manipulation in adult rats and the conditional D2 knockout mouse strains may reflect a developmental neuroadaptive increase in DAT activity to compensate for the hyperdopaminergia observed in these latter animals. There is strong evidence that the D2 autoreceptor and DAT interact physically and that this interaction facilitates trafficking of the DAT to the cell surface (Bolan et al., 2007; Eriksen et al., 2010; Chen et al., 2013). Disruption of physical D2-DAT interactions inhibits normal cell surface expression of the DAT (Lee et al., 2007). Furthermore, the DAT is known to be regulated in a variety of ways, including through βγ dimers (Garcia-Olivares et al., 2013) and a variety of interacting proteins (for review see Sager and Torres, 2011). Finally, we also examined the effect of D2R antagonism on DA kinetics in the dStr. Haloperidol administration under normal conditions (Garris et al., 2003), and in the SCR control animals (Figure 2), enhances electrically evoked DA release, an effect that has previously been attributed to antagonism of the D2 autoreceptor. Not surprisingly, the depletion of the D2R from the SN greatly attenuated the ability of haloperidol to enhance DA release in the dStr, and is an important pharmacological confirmation that the D2R was depleted from presynaptic terminals.

In terms of the behavioral consequences of the SN D2 knockdown, our rats displayed a phenotype characterized by hyperactivity, lack of habituation during open field exposure, and altered sensitivities to dopaminergic drugs. In many ways our model recapitulated the phenotype observed in the transgenic D2autoKO mice. For example, the SN D2 knockdown rats developed significant increases in locomotor activity within 2 weeks of virus infusion (Figure 4). This hyperactivity was long-lasting and progressive. Moreover, the hyperactivity was expressed in a familiar environment as the rats were repeatedly exposed to the open field chambers. However, unlike the DAT-D2autoKO mice, the SN D2 knockdown rats failed to show the typical within session habituation to the open field chamber. Interestingly, a similar lack of habituation has been observed in a DAT knockdown mouse, in which DAT levels are depleted to 10% of wildtype levels (Zhuang et al., 2001). The DAT knockdown mutants are hyperactive and have impaired response habituation, particularly in novel environments, a phenotype the authors attributed to an imbalance between pre- and post-synaptic D2 function. We observed a similar decrease in dopaminergic function in the SN D2 knockdown rats, where depletion of the SN D2R is associated with a decrease in *V*_max_ (Figure 3B), indicating that DAT function is reduced through a decrease in the amount of functional DAT (for review see Eriksen et al., 2010). The was no apparent change in either habituation or DAT function in DAT-D2autoKO mice. Together, the results from the DAT knockdown mutant mice and our D2 knockdown rats support the hypothesis that D2 autoreceptors in the dStr mediate habituation by alterations in DAT function.

The SN D2 knockdowns rats also displayed a decreased sensitivity to catalepsy induced by the atypical antipsychotic haloperidol (Figure 5A). In contrast, the Anazolone study demonstrated enhanced catalepsy in D2autoKO at a low dose of haloperidol, and the Bello et al D2autoKO mouse exhibited supersensitivity to haloperidol-induced inhibition of locomotor activity at relatively low doses. Blockade of the presynaptic D2 autoreceptor by haloperidol increases electrically evoked DA release (Figure 3), via the inhibition of autofeedback mechanisms (Garris et al., 2003). Indeed, our results clearly demonstrate that depletion of the SN D2R significantly decreases haloperidol-induced enhancement of electrically-evoked DA release (Figure 3). However, it is believed that the motoric side effects of haloperidol, such as catalepsy, are preferentially due to post-synaptic mechanisms (Klemm, 1985; Wadenberg et al., 2000). There are several possible explanations for decreased cataleptic activity in the SN D2 knockdown rats. Since activation of the D2 autoreceptor decreases DA synthesis and release (Budygin et al., 1999a; Joseph et al., 2002) and increases DA reuptake, the knockdown of the D2 could produce the opposite effects, resulting in an augmentation of dopaminergic tone. Under these conditions, more haloperidol may be needed to block this enhanced DA tone at post-synaptic D2R sites. Our behavioral results support this notion, as rats showed an increase in locomotor activity implying that the animals may be hyperdopaminergic. In addition, the FSCV results support a decrease in DA reuptake. However, the decrease in electrically-evoked DA release that does not suggest an augmentation in DA synthesis, while it cannot exclude an increase in basal DA efflux. Indeed, it is highly possible that the D2R removal from somatodendritic locations leads to an increased firing rate of DA neurons in the SN. We also cannot exclude a scenario in which the D2 knockdown is accompanied by compensatory alterations (increased sensitivity or density) in other presynaptic receptors, most notably the D3 receptor which has also been extensively implicated in the presynaptic regulation of DA release in the striatum (Gainetdinov et al., 1994, 1996; Joseph et al., 2002). This receptor has a much higher affinity for DA than the D2 DA receptor (Sokoloff et al., 1990), therefore, the decrease in electrically-evoked DA could be a consequence of the D3 receptor activation. Although the presynaptic D3 receptor appears to have a smaller role than D2 in regulating DA release under normal circumstances, it is possible that D3 could be upregulated or develop a more prominent role in the SN D2 knockdown rats.

The SN D2 knockdown rats also demonstrated altered responses to the locomotor stimulatory effects of cocaine. When normalized to baseline levels, the magnitude of the increase in locomotor activity among SN D2 knockdown rats after a high dose (20 mg/kg) of cocaine was in fact significantly less compared to SCR controls. These data are opposite to those observed in the D2autoKO transgenic mice, which exhibited an enhanced locomotor response to low and moderate doses of cocaine. The most probable explanation of these discrepancies is that deletion of D2 specifically from mesolimbic projections, but not nigrostriatal, contribute to the enhanced sensitivity to the locomotor effects of cocaine exhibited by the D2autoKO mice. There is evidence of functional selectivity in dopaminergic projections, with mesolimbic appearing to mediate locomotor effects of psychostimulants while nigrostriatal are more important for associated stereotypies (Amalric and Koob, 1993). In addition, it may be that the magnitude of effects of cocaine observed in these mouse models would not be as pronounced if the data were analyzed as a percentage of drug naïve or saline responses. Finally, since we used an escalating dose response, it is possible that a decrease in the development of cocaine sensitization in SN D2 knockdown rats may lead to this outcome. Regardless, our data highlight the importance of the D2 autoreceptor in setting the dynamic range and sensitivity to dopaminergic agents, even those that do not act directly through the D2R.

Perhaps the most surprising aspect of the acute depletion of SN D2R is the possible existence of neuroadaptations to the acute D2 knockdown. As discussed above, there may be compensatory expression or increased sensitivity of presynaptic D3 receptors as well as other possible adaptations, which could develop during a short period, including changes in the firing rate of DA cell bodies. However, this last one will be difficult to separate from the direct consequences of the D2 presynaptic DA receptor removal. Presumably similar mechanisms are at work in the transgenic mice as well, although undoubtedly alterations of these pathways in the developing animals can lead to additional compensatory alterations. Thus, our approach may be better suited for examining the development of both early and later adaptations, and could perhaps mimic adaptations of long term D2 antagonist drug treatments that are therapeutically applied in adolescence or adulthood. These results reinforce the need to understand the mechanisms underlying neuroadaptations that arise from manipulating DA neurotransmission.

## Author Contributions

EAB designed and supervised the voltammetry experiments, EBO collected voltammetry data, CEB, YBL, LCB and ACH performed the RNA analysis, MJB performed the histology, ACT designed the statistical analyses of the behavior and contributed to its interpretation, CEB designed the study and performed the behavioral assessments, and CEB and EAB wrote the manuscript, with contributions from ACT and ACH.

## Funding

This work was supported by the following NIH grants, K01-DA024863 (CEB), F31-DA032215 (LCB), F31-DA024525 (EBO), P50-DA006634 (ACH, CEB), R21-DA025321, R01-DA003690 (ACH), R01-AA022449 (EAB) and by the Russian Science Foundation grant N14-50-00069 (EAB).

## Conflict of Interest Statement

The authors declare that the research was conducted in the absence of any commercial or financial relationships that could be construed as a potential conflict of interest. The handling Editor declared a shared affiliation, though no other collaboration, with one of the authors EB and states that the process nevertheless met the standards of a fair and objective review.

## Abbreviations

*BACT*: beta-actin gene
*DRD2*: D2 dopamine receptor gene
[DA]_p_: DA release per pulse
dStr: dorsal striatum
*ENO2*: Enolase 2 gene
FSCV: fast scan cyclic voltammetry
SCR: scrambled.
